# The distortion of threshold approval matching

**DOI:** 10.1007/s10458-025-09724-6

**Published:** 2025-10-15

**Authors:** Mohamad Latifian, Alexandros A. Voudouris

**Affiliations:** 1 School of Informatics, University of Edinburgh, Scotland, UK; 2 School of Computer Science and Electronic Engineering, University of Essex, Colchester, UK

**Keywords:** Distortion, Matching problems, Approval, Social choice

## Abstract

We study matching settings in which a set of agents have private utilities over a set of items. Each agent reports a partition of the items into approval sets of different threshold utility levels. Given this limited information on input, the goal is to compute an assignment of the items to the agents (subject to cardinality constraints depending on the application) that (approximately) maximizes the social welfare (the total utility of the agents for their assigned items). We first consider the well-known, simple one-sided matching problem in which each of *n* agents is to be assigned exactly one of *n* items. We show that with *t* threshold utility levels, the distortion of deterministic matching algorithms is $\Theta (\root t \of {n})$ while that of randomized algorithms is $\Theta (\root t+1 \of {n})$. We then show that our distortion bounds extend to a more general setting in which there are multiple copies of the items, each agent can be assigned a number of items (even copies of the same one) up to a capacity, and the utility of an agent for an item depends on the number of its copies that the agent is given.

## Introduction

The assignment of papers to reviewers in conference management systems like CMT, HotCRP, EasyChair and OpenReview is computed using bidding information that classifies the papers into sets based on whether the reviewers are, for example, eager, willing, or not willing to handle them. In a sense, this process defines a collection of *threshold levels* that the reviewers (or, more generally, *agents*) can use to partition the papers (or, more generally, *items*) into associated *approval sets* based on their preferences (which can be dependent on their experience, their interests, and so on). Similar elicitation methods can be used in other important *constrained resource allocation* applications, such as the classic *house allocation* problem (and generalizations of it), where interested buyers (playing the role of agents) can bid for houses (corresponding to the items) by classifying them into categories depending on monetary value or other criteria.

Eliciting only threshold approvals rather than more detailed information about the underlying utility preferences of the agents for the items inevitably leads to inefficiency in terms of natural, cardinal objectives such as the well-known *social welfare* (the total utility). Typically, the loss of efficiency of decision-making methods that have access only to incomplete information is captured by the notion of *distortion*, which is defined as the worst-case ratio of the maximum possible social welfare over that of the computed solution. The distortion was originally used for social choice settings (such as voting) where decisions are made only based on ordinal information (rankings) [13, 31], but has recently been studied for settings in which different types of information is available or can be elicited (e.g., see [3, 4, 28–30]).

In the matching setting we study in this work, the threshold approvals reported by the agents is a type of information that lies in-between fully cardinal and fully ordinal since it provides some information about the range of the cardinal utilities and there is an order between items in different sets. Hence, while we cannot hope to achieve full efficiency, we can hope to achieve distortion better than what is possible just with ordinal preferences, depending on how detailed the threshold approvals are. In particular, we are interested in the possible *tradeoffs* between the distortion and the number of threshold levels both for when allocations are computed deterministically (which is the most natural way of doing so in social choice problems), as well as when randomization can be exploited.

### Our contribution

We start by considering the fundamental *one-sided matching problem* (also known as *house allocation*) to introduce the main ideas of our techniques, before turning to a more general setting. In one-sided matching, there is a set of *n* agents with utilities for a set of *n* items. The utilities are private and are not explicitly reported by the agents. Instead, for a number *t* of decreasing threshold values, each agent reports a collection of *t* *approval sets* consisting of items of different utility level; in particular, each approval set is associated with a threshold value and includes all items for which the agent has utility that is at least this threshold. Given the approval sets as input, our goal is to determine a one-to-one matching between agents and items so that the social welfare (total utility of the agents for their assigned items) is maximized.

We show tight bounds on the best possible distortion achieved by matching mechanisms for any number *t* of thresholds. In particular, we show a bound of $\Theta (\root t \of {n})$ for deterministic mechanisms, and a bound of $\Theta (\root t+1 \of {n})$ for randomized ones under the standard assumption of unit-sum utilities [13, 17].^1^ The lower bounds are presented in Section 3 and the upper bounds in Section 4. To put the bounds into perspective, we note that just one threshold is sufficient to obtain distortion $\Theta (n)$ for deterministic algorithms, beating the best possible distortion of $\Theta (n^2)$ that can be achieved using ordinal information [4]. Similarly, a distortion of $\Theta (\sqrt{n})$ can be achieved with randomization, matching the best possible distortion achieved by ordinal randomized algorithms [23].

Our deterministic mechanism is based on the following simple idea: Given the approval sets of the agents (induced by appropriately chosen threshold values), we compute a maximum-weight matching in a bipartite graph with the agents on one side, the items on the other side, and edges between them with weights equal to the lower threshold values associated with the approval sets. The slightly improved asymptotically tight randomized upper bound is obtained by running the deterministic mechanism with probability 1/2, while with the remaining 1/2 probability we compute a random matching (chosen equiprobably among all possible ones).

In Section 5, we turn our attention to a more general setting where the agents have *capacities*, indicating the maximum number of items they can receive, and the items have *supplies*, indicating the number of copies of them that are available. We make a budget-balance assumption that the total capacity is asymptotically of the same order of the total supply; for example, in the paper assignment problem, all papers must receive a (constant) number of reviews, and so the total capacity must be sufficiently larger than the total supply, but of the same magnitude. We also assume that, when agents can receive multiple copies of an item, their utility depends on the number of these copies, and thus the copies are not treated as independent items. Our goal is to compute an allocation of items to agents, such that the capacity and the supply constraints are satisfied, and the social welfare is maximized.

As this setting is a generalization of the one-sided matching (in which the number of agents is equal to the number of items, and there are unit capacities and supplies), our lower bounds from Section 3 extend directly. For the upper bounds, we show that, for *t* thresholds, the best possible distortion achieved by deterministic mechanisms is $O(c \cdot \root t \of {T})$, while the best distortion achieved by randomized mechanisms is $O(c \cdot \root t+1 \of {T})$, where $T$ is the total available supply (or capacity) and *c* is a parameter that depends either on the maximum capacity or the ratio between the number of items and agents. From this, we get bounds $\Theta (\root t \of {n})$ and $\Theta (\root t+1 \of {n})$ when the capacities and the supplies are constant.

### Related work

The distortion was originally defined by [31] to measure the worst-case loss in social welfare when voting decisions are made using only ordinal information. Since then, the distortion has been studied for several different voting problems, including utilitarian voting [13, 16, 17, 22, 31], metric voting [7, 18–20, 24, 27], and combinations of the two [25]. It has also been studied for social choice problems beyond voting, such as one-sided matching that we also consider in this paper [4, 5, 23], as well as other clustering and graph problems [1, 6, 14]. See the survey of [8] for an introduction to the distortion framework and the various models that have been considered.

Our paper follows a relatively recent stream of papers within the distortion literature that have considered elicitation methods beyond ordinal information. In this direction, [29, 30] showed tradeoffs between the best possible distortion and a communication complexity measure (the number of bits the agents can use to report information) for utilitarian voting. Results of similar flavour for metric voting have also been shown, for example, by [26].

More related to our work, in a series of papers, [3–5] studied voting and matching settings in which the agents provide ordinal information and, on top of that, are capable of answering *value queries* about their utilities for specific alternatives. They showed lower and upper bounds on the distortion of deterministic mechanisms that are functions of the number of queries per agent that are of similar to ours in the sense that the distortion decreases with the number of queries; some of their lower bounds (related to the number of queries required to achieve constant distortion) were recently improved by [15]. Another related paper is that of [28] who considered the one-sided matching problem when the agents can answer binary threshold queries about whether their utility for specific alternatives is larger than appropriately chosen thresholds. Using an approach similar to that of [4], they showed bounds on the distortion of deterministic mechanisms that is a function of the number of queries in terms of the social welfare among matchings that satisfy properties such as Pareto or rank-maximality. Ignoring differences in the models, the elicitation methods in these papers are related to the one we consider since the threshold approval sets can be computed using a number of (value or binary threshold) queries. Hence, our elicitation method is in a sense a bit more demanding. However, for the setting we focus here, we are able to show asymptotically tight bounds not only for deterministic mechanisms, but also for randomized ones, which have not been studied before.

Threshold approvals have also been recently explored in various other works on the distortion of voting mechanisms, most notably by [21] who showed that a single, appropriately chosen threshold is sufficient to achieve a distortion of $O(\sqrt{m})$ in utilitarian single-winner voting with *m* alternatives. In metric voting, [9] showed improved distortion bounds using an approval set per agent computed by a threshold value that is relative (rather than absolute) to the distance from the top-ranked alternative. Threshold approvals have also been considered in the context of participatory budgeting by [11], and voting under truthfulness constraints by [12]. All these works use just a single threshold, whereas we here explore the full potential of this elicitation method (for matching problems, rather than voting) using multiple thresholds and show tight bounds on the possible distortion.

## The one-sided matching problem

For any integer *t*, let $[t] = \{1, \dots , t\}$. We start with the simple *one-sided matching* setting to express the core idea; in Section 5, we show that our results extend to a more general setting that more accurately captures applications such as paper assignment in peer reviewing. Let $\mathcal {N}$ be a set of *n* *agents* and $\mathcal {M}$ be a set of *n* *items*. Agent *i* has a *utility function* $u_i: \mathcal {M}\rightarrow [0, 1]$ over the items. We assume that these utility functions satisfy the unit-sum assumption, which means for each agent $i \in \mathcal {N}$, $\sum _{j \in \mathcal {M}} u_i(j) = 1$. Together, these utility functions form the *utility profile *$\vec {u}$. A *matching* of the items to the agents is a bijection $A: \mathcal {N}\rightarrow \mathcal {M}$. With a slight abuse of notation, we use $A_i = A(i)$ to refer to the item matched to agent *i*, and also *A*(*a*) to refer to the agent matched to item *a*. We define the *social welfare* of a matching $A$ under utility profile $\vec {u}$ to be the total utility of the agents for the items they are matched to, i.e.,

$$\begin{aligned} \textsf{sw}(A, \vec {u}) = \sum _{i \in \mathcal {N}} u_i(A_i) = \sum _{a \in \mathcal {M}} u_{A(a)}(a). \end{aligned}$$

The goal is to compute a matching with high social welfare in the worst case. For ease of notation, we will drop $\vec {u}$ from $\textsf{sw}(A,\vec {u})$ whenever it is clear from context.

### Elicitation method

In this paper, we focus on eliciting threshold approval votes with a *threshold vector* $\vec {\tau }= (\tau _1, \ldots , \tau _t)$ such that $\tau _1> \ldots > \tau _t$. Given such a vector we ask each agent *i* to submit *t* disjoint *threshold approval subsets* of $\mathcal {M}$, denoted by $S_{i,1}, \ldots , S_{i,t}$, where $S_{i,k}$ includes the items for which the agent has utility in [$\tau _{k-1}, \tau _k)$, with $\tau _0:= 1$. In other words, $S_{i, k} = \{j \in \mathcal {M}:\tau _{k-1} \ge u_i(j) > \tau _k\}$. These sets do not necessarily partition $\mathcal {M}$ as there might exit item *j* for which $u_i(j) < \tau _t$. All these $n \times t$ threshold approval sets form the *input profile* $\textbf{S}$. Note that different utility profiles might induce the same input profile. We say that a utility profile $\vec {u}$ is consistent with an input profile $\textbf{S}$ (in which say we write $\vec {u}\rhd \textbf{S}$) if for each agent $i\in \mathcal {N}$, $k \in [t]$, and $j \in S_{i, k}$, $\tau _{k-1}\ge u_i(j) > \tau _k$.

### Mechanisms and distortion

A *mechanism* *f* defines a threshold vector $\vec {\tau }$, takes an input profile $\textbf{S}$ based on $\vec {\tau }$, and then outputs a matching $f(\textbf{S})$ of the items to the agents. The distortion of a matching $A$ on input profile $\textbf{S}$ is defined as:

$$\begin{aligned} \textsf{dist}(A, \textbf{S}) = \sup _{\vec {u}\rhd \textbf{S}} \frac{\textsf{sw}(\mathsf {A^*}, \vec {u})}{\textsf{sw}(A, \vec {u})}, \end{aligned}$$

where $\mathsf {A^*}$ is the matching with the maximum social welfare with respect to $\vec {u}$. The distortion of a matching mechanism *f* is defined as the worst case distortion of *f* on any input profile:

$$\begin{aligned} \textsf{dist}(f) = \sup _{\textbf{S}} \textsf{dist}(f(\textbf{S}), \textbf{S}). \end{aligned}$$

## Lower bounds

In this section we show lower bounds on the best possible distortion achievable by deterministic and randomized mechanisms for the one-sided matching problem. In particular, for mechanisms that use $t \ge 1$ thresholds, we show a lower bound of $\Omega (\root t \of {n})$ for deterministic mechanisms and a lower bound of $\Omega (\root t+1 \of {n})$ for randomized mechanisms. We start by showing a technical lemma that holds for randomized mechanisms that will be useful in establishing the lower bounds in several cases. For a randomized mechanism *f*, denote by *p*(*i*, *a*) the probability that item *a* is assigned to agent *i* according to *f*.

### Lemma 3.1

For any subset of items $M \subseteq \mathcal {M}$, let $A_M$ be the matching of the items in *M* to the agents with minimum sum of probabilities over the items matched to them in $A_M$ with respect to *f*. Then, $\sum _{a \in M} p(A_M(a),a) \le 1.$

### Proof

We will prove the claim by constructing a matching *A* with sum of probabilities at most 1; then, since $A_M$ is the matching with minimum sum of probabilities, the same must hold for it as well. Consider the following greedy algorithm: Starting with $T = M$ and $N = \mathcal {N}$, in each iteration, find the pair $(i,a) \in N \times T$ with the minimum possible *p*(*i*, *a*), assign *a* to *i*, and then remove *a* and *i* from *T* and *N*, respectively.

Let $P_j$ be the sum of probabilities of the assigned items at the end of the *j*-th iteration. We will show by induction that $P_j \le j/|M|$. For $j=1$, by the pigeonhole principle, we can find a pair with $p(i, a) \le 1/|M|$. So, we now assume that our hypothesis holds for any $j < \ell$, and want to prove that $P_\ell \le \ell / |M|$. Let (*i*, *a*) be a pair with minimum *p*(*i*, *a*) in the $\ell$-th iteration. Since the algorithm is greedy, we know that for any item *b* that was previously assigned to agent *A*(*b*), $p(A(b), b) \le p(i, b)$. This means that the sum of the probabilities of *i* receiving any item assigned in a previous iteration is at least $P_{\ell -1}$. Hence, by the pigeonhole principle again, $p(i, a) \le \frac{1-P_{\ell -1}}{|M|-\ell +1}$, which implies that

$$\begin{aligned} P_\ell = P_{\ell -1} + p_{i, a}&\le P_{\ell -1} + \frac{1-P_{\ell -1}}{|M|-\ell +1} \\&\le \frac{\ell -1}{|M|}+\frac{1-\frac{\ell -1}{|M|}}{|M|-\ell +1} = \frac{\ell -1}{|M|}+\frac{\frac{|M|-\ell +1}{|M|}}{|M|-\ell +1} = \frac{\ell }{|M|}. \end{aligned}$$

Consequently, we overall have that $\sum _{a \in M} p(A_M(a),a) \le P_{|M|} \le 1$. $\square$

We are now ready to show the lower bounds via a sequence of lemmas capturing different cases. The first lower bound depends on the ratio of consecutive threshold levels and holds for any mechanism (randomized or deterministic).

### Lemma 3.2

Consider a threshold vector $\vec {\tau }= (\tau _1, \ldots , \tau _t)$, and let $k \in [t]$ be such that $\delta = \tau _{k-1}/\tau _{k}$ is the largest multiplicative gap between two consecutive thresholds (assuming $\tau _0 = 1$). Then, the distortion of any matching mechanism *f* that uses $\vec {\tau }$ is $\Omega (\delta )$.

### Proof

For constant $\delta$ the bound is trivial, so consider $\delta > 2$. For an infinitesimal $\varepsilon$, define $\tau _k^+:= \tau _k + \varepsilon$, and find integer *m* such that $\tau _{k-1}/2 + m\tau _k^+ \le 1 < \tau _{k-1}/2 + (m+1)\tau _k^+$. We partition the items into three sets: $\mathcal {M}_1$ with $m+1$ items, $\mathcal {M}_2$ with 1 item, and $\mathcal {M}_3$ with $n-m-2$ items. Note that since $\varepsilon$ is an infinitesimal, we have

1 $$\begin{aligned} \tau _{k-1}/2 + (m+1)\tau _k^+> 1 \implies (m+1)\tau _k^+> 1/2 \implies m+1 > \frac{\delta }{2}. \end{aligned}$$

Now consider an instance with input profile such that

- $$S_{i,k} = \mathcal {M}_1$$,
- $S_{i,k'} = \mathcal {M}_2$ for some $k' > k$, and
- $$S_{i,j} = \emptyset $$ for $$j \in [t] \setminus \{ k, k' \}$$

We define the following consistent utility profile (that is, the utilities of each agent sum up to 1 and the utilities induce the aforementioned input profile). Let $A_{\mathcal {M}_1}$ be the matching of the items in $\mathcal {M}_1$ to the agents with minimum sum of probabilities, and let $\mathcal {N}_1$ be the set of agents that are assigned an item from $\mathcal {M}_1$ according to the matching $A_{\mathcal {M}_1}$, that is, $\mathcal {N}_1:= \{ i \in \mathcal {N}: \exists a \in \mathcal {M}_1, A_{\mathcal {M}_1}(a) = i \}$. For fixed item $a^* \in \mathcal {M}_1$, we define the utility function

$$u_i(a) = {\left\{ \begin{array}{ll} \tau _{k-1}/2 & a \in \mathcal {M}_1, A_{\mathcal {M}_1}(a) = i \\ \tau _{k-1}/2 & a = a^*, i \notin \mathcal {N}_1 \\ 1-\tau _{k-1}/2-m\tau _k^+ & a \in \mathcal {M}_2 \\ 0 & a \in \mathcal {M}_3\\ \tau _{k}^+ & o.w. \end{array}\right. }$$

Observe that, for any agent *i*, the utilities sum up to 1. For any agent *i* there is exactly one item in $\mathcal {M}_1$ for which *i* has value $\tau _{k-1}/2$ (either $i \in \mathcal {N}_1$ and thus there is a single item $a \in \mathcal {M}_1$ such that $A_{\mathcal {M}_1}(a)=i$ for which *i* has value $\tau _{k-1}/2$, or $i \not \in \mathcal {N}_1$ and thus *i* has value $\tau _{k-1}/2$ for $a^*$), and $|\mathcal {M}_1|-1 = m$ items for which *i* has value $\tau _k^+$. Consequently,

$$\begin{aligned} \sum _{a \in \mathcal {M}} u_i(a) = \frac{\tau _{k-1}}{2} + m \tau _k^+ + 1- \frac{\tau _{k-1}}{2}-m\tau _k^+ = 1. \end{aligned}$$

Also, observe that the utility function is consistent to the input profile: for any item $a \in M_1$, the utility of agent *i* is either $\tau _{k-1}/2$ or $\tau _k^+$, i.e., in the interval $[\tau _{k-1},\tau _k)$; for the single item $a \in M_2$, by the choice of *m*, the utility of agent *i* is

$$\begin{aligned} 1-\frac{\tau _{k-1}}{2}-m\tau _k^+ < \frac{\tau _{k-1}}{2}+(m+1)\tau _k^+ -\frac{\tau _{k-1}}{2}-m\tau _k^+ = \tau _k^+ = \tau _k + \varepsilon . \end{aligned}$$

Since the inequality is strict, the utility is at most $\tau _k$ for infinitesimal $\varepsilon$.

Now consider the expected social welfare of *f*(*S*) when $\varepsilon \rightarrow 0$ according to the above utility profile. The maximum achieved utility is $\tau _{k-1}/2$ from $a^*$,

$$\begin{aligned} p(A_{\mathcal {M}_1}(a), a)\cdot \tau _{k-1}/2 + (1-p(A_{\mathcal {M}_1}(a), a))\cdot \tau _k \end{aligned}$$

from each item $a\in \mathcal {M}_1 \setminus \{a^*\}$, and at most $\tau _k$ from the item in $\mathcal {M}_2$. By Lemma 3.1, we have that $\sum _{a \in \mathcal {M}_1} p(A_{\mathcal {M}_1}(a), a) \le 1$, and thus

$$\begin{aligned} {{\,\mathrm{\mathbb {E}}\,}}_{A\sim f(S)}[\textsf{sw}(A, \vec {u})]&\le \frac{\tau _{k-1}}{2} + \sum _{a \in \mathcal {M}_1} \bigg ( p(A_{\mathcal {M}_1}(a), a) \cdot \left( \frac{\tau _{k-1}}{2}-\tau _k \right) + \tau _k \bigg ) + \tau _k \\&\le \tau _{k-1} + (m+1) \tau _k. \end{aligned}$$

In the optimal matching, we can assign each item $a \in \mathcal {M}_1$ to agent $A_{\mathcal {M}_1}(a)$ for a social welfare of $(m+1) \tau _{k-1}/2$. Due to (1), the distortion is

$$\begin{aligned} \frac{1}{2} \cdot \frac{(m+1)\tau _{k-1}}{\tau _{k-1} + (m+1)\tau _k}&\ge \min \left( \frac{m+1}{2}, \frac{\tau _{k-1}}{2\tau _k}\right) \in \Omega (\delta ). \end{aligned}$$

and the proof is complete. $\square$

Our next two lemmas provide lower bounds for deterministic and randomized mechanisms, respectively, for when the last threshold level is sufficiently large.

### Lemma 3.3

Consider a threshold vector $\vec {\tau }= (\tau _1, \ldots , \tau _t)$ such that $\tau _t \ge 1/(n-1)$. Then, the distortion of any deterministic matching mechanism *f* that uses $\vec {\tau }$ is unbounded.

### Proof

Consider the input profile $\textbf{S}$ where the threshold approval sets of any agent are empty, and thus the utility of any agent for any item is at most $\tau _t$. Let $A=f(\textbf{S})$ be the matching computed by the deterministic matching mechanism *f*, and let *B* be another matching such that $A(a) \ne B(a)$ for every item $a \in \mathcal {M}$. Consider the utility profile $\vec {u}$ where agents have utility 0 for their matched item in *A*, utility $\tau _t$ for their matched item in *B*, and $(1-\tau _t)/(n-2)$ for each of the remaining $n-2$ items. Note that $\tau _t \ge 1/(n-1) \implies (1-\tau _t)/(n-2) \le \tau _t$, and hence $\vec {u}\rhd \textbf{S}$. Since $\textsf{sw}(A,\vec {u}) = 0$ and $\textsf{sw}(B,\vec {u}) = n \cdot \tau _t > 0$, the distortion is unbounded. $\square$

### Lemma 3.4

Consider a threshold vector $\vec {\tau }= (\tau _1, \ldots , \tau _t)$ such that $\tau _t > 1/n$. Then, the distortion of any randomized matching mechanism *f* that uses $\vec {\tau }$ is $\Omega (n\cdot \tau _t)$.

### Proof

Consider the input profile $\textbf{S}$ where the threshold approval sets of any agent are empty, and thus the utility of any agent for any item is at most $\tau _t$. Let $A_\mathcal {M}$ be the matching over $\mathcal {M}$ with minimum sum of probabilities; by Lemma 3.1, $\sum _{a \in \mathcal {M}} p(A_\mathcal {M}(a), a) \le 1$. Now, consider the utility profile $\vec {u}$ where each agent has utility $\tau _t$ for the item she is matched to according to $A_{\mathcal {M}}$ and utility $(1-\tau _t)/(n-1)$ for each of the remaining items. Note that $\tau _t \ge 1/n \implies (1-\tau _t)/(n-1) \le \tau _t$, and hence $\vec {u}\rhd \textbf{S}$. The expected social welfare of the mechanism is

$$\begin{aligned} {{\,\mathrm{\mathbb {E}}\,}}_{A \sim f(S)}\left[ \sum _{a \in \mathcal {M}} u_{A(a)}(a)\right]&= \sum _{a \in \mathcal {M}} \bigg ( p(A_\mathcal {M}(a), a) \cdot \tau _t + (1- p(A_\mathcal {M}(a), a) \cdot \frac{1-\tau _t}{n-1} \bigg ) \\&= \left( \tau _t - \frac{1-\tau _t}{n-1} \right) \sum _{a \in \mathcal {M}} p(A_\mathcal {M}(a), a) + n \cdot \frac{(1-\tau _t)}{n-1} \\&\le \tau _t - \frac{1-\tau _t}{n-1} + n \cdot \frac{(1-\tau _t)}{n-1} = 1. \end{aligned}$$

Since $\textsf{sw}(A_{\mathcal {M}},\vec {u}) = n\cdot \tau _t$, the distortion is at least this much. $\square$

By appropriately combining Lemmas 3.2, 3.3, and 3.4, we can establish the desired lower bounds on the distortion of the different types of mechanisms.

### Theorem 3.5

The distortion of any deterministic matching mechanism *f* that uses a threshold vector $\vec {\tau }$ of length *t* is $\Omega (\root t \of {n})$.

### Proof

If $\tau _t \ge 1/(n-1)$, by Lemma 3.3, the distortion is unbounded. Otherwise, if $\tau _t \le 1/(n-1)$, let $k \in \arg \max _{j \in [t]} \tau _{j-1}/\tau _j$ with $\tau _0 = 1$. Clearly,

$$\begin{aligned} \bigg ( \frac{\tau _{k-1}}{\tau _k} \bigg )^t \ge \prod _{j \in [t]} \frac{\tau _{j-1}}{\tau _j} = \frac{1}{\tau _t} \implies \delta = \frac{\tau _{k-1}}{\tau _k} \ge \tau _t^{-1/t} \ge \root t \of {n}, \end{aligned}$$

and thus, by Lemma 3.2, the distortion is $\Omega (\root t \of {n}).$ $\square$

### Theorem 3.6

The distortion of any matching mechanism *f* that uses a threshold vector $\vec {\tau }$ of length *t* is $\Omega (\root t+1 \of {n})$.

### Proof

Suppose that the threshold vector $\vec {\tau }$ is such that $\tau _t > n^{-t/(t+1)}$. Since $n^{-t/(t+1)} \ge n^{-1}$, by Lemma 3.4, the distortion of *f* is $\Omega (n\cdot \tau _t) = \Omega (\root t+1 \of {n})$. So, we can now assume that $\tau _t \le n^{-t/(t+1)}$. As in the proof of Theorem 3.5, we have that $\delta = \tau _{k-1} / \tau _k \ge \tau _t^{-1/t} \ge \root t+1 \of {n}$, and thus, by Lemma 3.2, the distortion of *f* is $\Omega (\delta ) = \Omega (\root t+1 \of {n})$. $\square$

## Upper bounds

In this section we present asymptotically tight upper bounds for deterministic and randomized matching mechanisms. Our deterministic mechanism (described below) computes a maximum-weight matching by assuming that each agent has the minimum possible utility (according to the thresholds) for all the items in the different approval set given as input.

### Definition 4.1

For $\delta > 1$ and $t \in [n]$, we define the threshold vector $\vec {\tau }= (\delta ^{-1}, \delta ^{-2}, \ldots , \delta ^{-t})$. The *deterministic matching mechanism* $f_t$ uses this threshold vector $\vec {\tau }$ and, given an input profile $\textbf{S}$, constructs the following weighted bipartite graph $G_\textbf{S}$: There are 2*n* nodes in total, consisting of a node $v_i$ for each agent $i \in \mathcal {N}$ on the left side and a node $z_a$ for each item $a \in \mathcal {M}$ on the right side. For $i \in \mathcal {N}$, $k \in [t]$ and $a \in S_{i,k}$, there is an edge from $v_i$ to $z_a$ with weight $w(v_i,z_a)=\tau _k$. The mechanism $f_t$ finds the maximum weighted matching in $G_\textbf{S}$ and, for each matched pair $(v_i, z_a)$, assigns item *a* to agent *i*. If there are unmatched pairs remaining, $f_t$ completes the allocation arbitrarily.

### Example 4.2

**Fig. 1:**
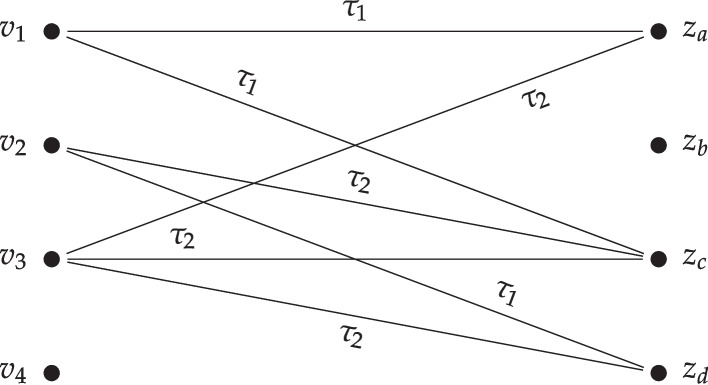
The graph $G_\textbf{S}$ that is used by $f_t$ in Example 4.2

Let $t=2$ and $\vec {\tau }= (\tau _1, \tau _2)$. Suppose that $S_{1,1} = \{ a,c \}$, $S_{2,1}=\{ d \}, S_{2,2}=\{ c \}, S_{3,2}=\{ a, c, d \}$, while the remaining approval sets are empty. Mechanism $f_t$ constructs the graph $G_\textbf{S}$ shown in Fig. 1, computes a maximum-weight matching, and then assigns any unmatched items arbitrarily.

Before we bound the distortion of the mechanism, we prove two very useful technical lemmas. The first one provides us with a lower bound on the weight of the maximum-weight matching in a bipartite graph whose nodes satisfy certain properties; this will be used extensively to lower bound the social welfare of the matching computed by $f_t$, and also by the deterministic mechanism in Section 5.

### Lemma 4.3

Consider a weighted bipartite graph *G* with *n* nodes on the left side $\{v_1, \ldots , v_n\}$ and *m* nodes $\{z_1, \ldots , z_m\}$ on the right side. If $\sum _{a \in [m]} w(v_i, z_a) \ge W$ for each $v_i$, and $w(v_i, z_a) \ge L$ for each edge $(v_i, z_a)$, then there is matching in *G* with weight at least $\min \{W, nL\}$.

### Proof

We construct a matching *B* in *G* as follows: For every $a \in [m]$, we match node $z_a$ to a node $v_i$ such that the edge $(v_i,z_a)$ exists in *G* and $w(v_i,z_a)$ is the maximum possible among all available (not already matched) nodes in $\{v_1, \ldots , v_n\}$. If all nodes in $\{v_1, \ldots , v_n\}$ are matched to a node in $\{z_1, \ldots , z_m\}$, then since the weight of any edge is at least *L*, the total weight of *B* is at least *nL*. Otherwise, if there exists node $v_i$ that is not matched to any node in $\{z_1, \ldots , z_m\}$ according to *B*, then by the definition of *B*, $w(v_{B(z_a)},z_a) \ge w(v_i,z_a)$ for any $z_a$, and hence the weight of *B* is

$$\begin{aligned} \sum _{a \in [m]} w(v_{B(z_a)},z_a) \ge \sum _{a \in [m]} w(v_i,z_a) \ge W. \end{aligned}$$

Consequently, overall, the weight of *B* is at least $\min \{W, nL\}$. $\square$

The second lemma shows that $G_\textbf{S}$ admits a matching with weight that is relatively close to the social welfare of the optimal matching allocation for any utility profile consistent to the input profile $\textbf{S}$. We will use this relation in the analysis of the distortion of $f_t$, as well as in the analysis of our randomized matching mechanism later on.

### Lemma 4.4

Let $\mathsf {s^*}$ be the social welfare of the optimal matching allocation. There exists a matching of $G_\textbf{S}$ with weight at least $\delta ^{-1}(\mathsf {s^*}- n\tau _t)$.

### Proof

For any agent *i*, let $a^*_i$ be the item that *i* is given in the optimal matching allocation $A^*$. Clearly, either there exists $j \in [t]$ such that $a_i^* \in S_{i,j}$, or $a_i^* \not \in \bigcup _{j \in [t]} S_{i,j}$. The total utility accumulated by the agents of the second type is at most $n\tau _t$. For the agents of the first type, since $a_i^* \in S_{i,j}$, there is an edge between $v_i$ and $z_{a_i^*}$ in $G_\textbf{S}$ of weight

$$\begin{aligned} \tau _j = \delta ^{-j} = \delta ^{-1}\cdot \delta ^{-j+1} = \delta ^{-1}\cdot \tau _{j-1} \ge \delta ^{-1} \cdot u_i(a_i^*). \end{aligned}$$

Hence, the intersection of $A^*$ and $G_\textbf{S}$ gives us a matching of weight at least $\delta ^{-1}(\mathsf {s^*}- n\tau _t)$. $\square$

### Theorem 4.5

For $t \in [n]$ and $\delta = \root t \of {2n}$, the distortion of the deterministic matching mechanism $f_t$ is $O(\root t \of {n})$.

### Proof

Let $A = f_t(\textbf{S})$ be the matching computed by the mechanism $f_t$ when given as input an arbitrary input profile $\textbf{S}$ that is induced by some consistent utility profile $\vec {u}$. First, observe that if $G_\textbf{S}$ admits a matching of weight *W* then $\textsf{sw}(A,\vec {u}) \ge W$. This follows by the fact that if node $v_i$ is matched to node $z_a$ in $G_\textbf{S}$, then agent *i* has utility at least $w(v_1,z_a)$ for item *a*.

Second, we argue that the weight of the maximum weight matching of $G_\textbf{S}$ is at least $\delta ^{-1}/2$. The total utility of an agent for the items in $\bigcup _{j \in [t]} S_{i,j}$ is at least 1/2 since the utility for each of the remaining items is at most $\tau _t = \delta ^{-t} = 1/(2n)$. Since $\tau _{j-1} = \tau _j \cdot \delta$ with $\tau _0 = 1$, and $v_i(a) \le \tau _{j-1}$ and $w(v_i,z_a) = \tau _j$ for every $a \in S_{i,j}$, we have

$$\begin{aligned}&\frac{1}{2} \le \sum _{j \in [t]} \sum _{a \in S_{i,j}} v_i(a) \le \sum _{j \in [t]} |S_{i,j}| \tau _{j-1} = \delta \cdot \sum _{j \in [t]} |S_{i,j}| \tau _j \\&\implies \sum _{j \in [t]} \sum _{a \in S_{i,j}} w(v_i,z_a) \ge \frac{\delta ^{-1}}{2}. \end{aligned}$$

Since the edge $(v_i,z_a)$ exists in $G_\textbf{S}$ only if $a \in \bigcup _{j \in [t]} S_{i,j}$, we have that $\sum _{a \in \mathcal {M}} w(v_i,z_a) \ge \delta ^{-1}/2$ and $w(v_i,z_a) \ge \tau _t = 1/(2n)$, and thus, by Lemma 4.3, we have that $G_\textbf{S}$ admits a matching of weight at least $\min \{\delta ^{-1}/2,1/2\}$, which is equal to $\delta ^{-1}/2$ since $\delta > 1$. By Lemma 4.4, since $n\tau _t = 1/2$, we also have that $G_\textbf{S}$ admits a matching of weight at least $\delta ^{-1}(\mathsf {s^*}- 1/2)$, where $\mathsf {s^*}$ is the optimal social welfare. Consequently, we overall have established that the social welfare of the allocation *A* computed by the mechanism is

$$\begin{aligned} \textsf{sw}(A) \ge \max \bigg \{ \frac{1}{2} \cdot \delta ^{-1}, \delta ^{-1}(\mathsf {s^*}- 1/2) \bigg \}. \end{aligned}$$

Hence, the distortion is at most

$$\begin{aligned} \frac{\delta \cdot \mathsf {s^*}}{\max \{1/2, \mathsf {s^*}- 1/2\}} \end{aligned}$$

If $\mathsf {s^*}\le 1$, then the distortion is at most $2\delta \mathsf {s^*}\le 2\delta$. Otherwise, if $\mathsf {s^*}\ge 1$, then $\mathsf {s^*}- 1/2 \ge \mathsf {s^*}/2$ and the distortion is at most $\delta \cdot \frac{\mathsf {s^*}}{\mathsf {s^*}/2} = 2 \delta$. In any case, the distortion is at most $2\delta \in O(\root t \of {n}).$ $\square$

Due to Theorem 4.5, we can achieve linear distortion using a single threshold level (which is a significant improvement compared to the $O(n^2)$ distortion that can be achieved with ordinal information) and constant distortion using a logarithmic number of threshold levels.

### Corollary 4.6

We can achieve distortion *O*(*n*) by using one threshold level and distortion *O*(1) by using $t = O(\log {n})$ threshold levels.

We now turn our attention to randomization. We design a mechanism that is a convex combination of the naive rule which chooses a random matching equiprobably among all possible ones, and the deterministic matching mechanism $f_t$ that was analyzed above.

### Definition 4.7

The *randomized matching mechanism* $R_t$ with probability 1/2 chooses a matching uniformly at random, and with probability 1/2 runs the deterministic mechanism $f_t$ with threshold vector $\vec {\tau }= (\delta ^{-1}, \delta ^{-2}, \ldots , \delta ^{-t})$ for $t \in [n]$ and some $\delta > 1$.

### Theorem 4.8

For $t \in [n]$ and $\delta = \root t+1 \of {n}$, the distortion of the randomized matching mechanism $R_t$ is $O(\root t+1 \of {n})$.

### Proof

Let $A^*$ be an optimal matching with social welfare $s^*$, and $A^2$ the matching computed by the second part of the mechanism (the outcome of $f_t$). In the first part of the mechanism (where a random matching is chosen with probability 1/2), since each possible matching has probability at least 1/(*n*!), each agent is matched to each item with probability at least 1/*n*. Since the sum of the utilities of each agent for all items is 1, the expected social welfare from the first part is at least

$$\begin{aligned} \frac{1}{2} \sum _{i \in \mathcal {N}} \sum _{a \in \mathcal {M}} \frac{1}{n} \cdot u_i(a) = \frac{1}{2} \cdot n \cdot \frac{1}{n} = \frac{1}{2}. \end{aligned}$$

For the second part of the mechanism (where the deterministic mechanism $f_t$ using $\vec {\tau }$ is employed), since $s:= n \tau _t = n \delta ^{-t} = \root t+1 \of {n}$, by Lemma 4.4, there is a matching in $G_\textbf{S}$ of weight at least $\delta ^{-1}(\mathsf {s^*}- s)$, and thus the expected social welfare of the mechanism from the second part is at least $\frac{1}{2} \cdot \delta ^{-1}(\mathsf {s^*}- s)$. Overall, we have established that

$$\begin{aligned} {{\,\mathrm{\mathbb {E}}\,}}_{A\sim R_t(\textbf{S})}[\textsf{sw}(A)]&\ge \frac{1}{2} + \frac{1}{2} \cdot \delta ^{-1}(\mathsf {s^*}- s) \\&\ge \frac{1}{2} \cdot \max \bigg \{ 1, \delta ^{-1}(\mathsf {s^*}- s) \bigg \}, \end{aligned}$$

and thus the distortion is at most

$$\begin{aligned} \frac{2 \cdot \mathsf {s^*}}{\max \{1, \delta ^{-1}(\mathsf {s^*}- s)\}}. \end{aligned}$$

If $\mathsf {s^*}\ge 2s$, then $\mathsf {s^*}- s \ge \mathsf {s^*}/2$ and the distortion is at most $\frac{2 \cdot \mathsf {s^*}}{\delta ^{-1}\mathsf {s^*}/2} = 4\delta = 4\root t+1 \of {n}.$ Otherwise, if $\mathsf {s^*}< 2s$, the distortion is at most $2\mathsf {s^*}\le 4s = 4\root t+1 \of {n}.$ In any case, the distortion $O(\root t+1 \of {n})$. $\square$

## Generalized setting

In this section we consider a generalized setting. Similarly to before, $\mathcal {N}$ represents a set of $n \ge 1$ agents. However, here it is not necessarily the case that we have an equal number of items; we define $\mathcal {M}$ to be a set of $m \ge 1$ items. Each item $a \in \mathcal {M}$ has a supply $m_a \ge 1$, and each agent $i \in \mathcal {N}$ has a capacity $c_i \ge 1$. For simplicity, we assume that the total supply is equal to the total capacity, that is, $T:= \sum _{i \in \mathcal {N}} c_i = \sum _{a \in \mathcal {M}} m_a$.^2^ While our results hold for any capacity, to make our results interpretable, we will pay particular focus on the case of constant capacities at the end of the proof.

Agents are allowed to receive copies of the same item, in which case their utility depends on the number of copies they receive; in other words, copies of an item are not considered independent. For each agent *i*, item *a* and $j \in [\min \{c_i, m_a\}]$, we denote by $u_i(a,j)$ the *marginal utility *that agent *i* gets when receiving his *j*-th copy of item $a \in \mathcal {M}$, and by $u^+_i(a,j)$ his *total utility* when receiving $j \le \min \{c_i, m_a\}$ copies of item $a \in \mathcal {M}$, i.e., $u^+_i(a,j) = \sum _{k \in [j]} u_i(a,j)$.

An *allocation* $X = (x_i(a))_{i \in \mathcal {N}, a \in \mathcal {M}}$ determines the number $x_i(a)$ of copies of item *a* that agent *i* is assigned to, such that $\sum _{a \in \mathcal {M}} x_i(a) \le c_i$ for every $i \in \mathcal {N}$ and $\sum _{i \in \mathcal {N}} x_i(a) \le m_a$ for every $a \in \mathcal {M}$. Given an allocation *X*, the utility of *i* for *X* is $u_i(X) = \sum _{a \in \mathcal {M}} u^+_i(a,x_i(a))$. We assume that the utility function of each agent *i* satisfies the unit-sum assumption, that is, $\sum _{a \in \mathcal {M}} u^+_i(a, \min \{c_i, m_a\}) = 1$. The definition of the social welfare of an allocation is the same as before, that is, it is the total utility of the agents for the allocation. We aim to compute allocations with high social welfare that maximally assign the items to the agents; observe that the maximum possible number of items that can be allocated is $T$ (or $\min \left\{ \sum _{i \in \mathcal {N}} c_i, \sum _{a\in \mathcal {M}} m_a\right\}$ in the more general case), and that any allocation *Y* that assigns less than $T$ items is dominated in terms of social welfare by any allocation *X* that assigns the items allocated by *Y* in the same way, but also somehow assigns the remaining items. So, in the following, we focus on such maximal allocations only.

Since the utility functions depend on the number of item copies that the agents receive, we need to appropriately redefine the *elicitation method*. For a threshold vector $\vec {\tau }= (\tau _1, \ldots , \tau _t)$, each agent *i* reports *t* disjoint threshold approval sets $S_{i,1}, \ldots , S_{i,t}$, where $S_{i,k}$ includes pairs of items and indices for which *i* has marginal utility in [$\tau _{k-1}, \tau _k)$, where $\tau _0:= 1$. In other words, $S_{i, k} = \{a \in \mathcal {M}, j \in [\min \{c_i,m_a\}] :\tau _{k-1} \ge u_i(a, j) > \tau _k\}$. The input profile $\textbf{S}$ now consists of these threshold approval sets reported by all agents. The definition of distortion can also be appropriately refined by taking the worst case over utility profiles and input profiles consistent with them.

We now define a parametric min-cost flow instance that will be used by our algorithms later.

### Definition 5.1

**Fig. 2:**
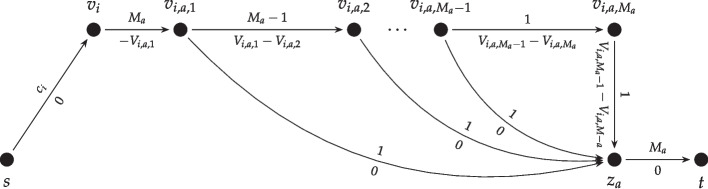
Graph component between each pair $(i, a) \in \mathcal {N}\times \mathcal {M}$

(Min-cost Flow Instance) Let $C = (c_i)_{i \in \mathcal {N}}$ be a capacity vector of the *n* agents with maximum value *c*, $M = (M_a)_{a \in \mathcal {M}}$ be a supply vector of the *m* items, and $V_{n\times m \times c}$ a value matrix in which *V*(*i*, *a*, *j*) is the value of agent *i* when receiving *j* copies of item *a*. Let *G*(*C*, *M*, *V*) be the following min-cost flow instance: *G* has a source node *s* and a destination node *t*. Furthermore, there is a node $v_i$ for each agent *i* and a node $z_a$ for each item *a*. For every *i*, there is an edge $(s,v_i)$ with capacity $c_i$ and cost 0. For every *a*, there is an edge $(z_a,t)$ with with capacity $M_a$ and cost 0. For each agent *i* and item *a*, we add a component to the graph as shown in Fig. 2. Nodes *s* and *t* have supply and demand equal to $\min \left( \sum _{i \in [n]} c_i,\sum _{a \in [m]} M_a\right)$, respectively. The goal is to find the minimum cost for satisfying this flow.

In the described network, each unit of flow represents one item. The capacity of the edge $(s, v_i)$ ensures that agent *i* gets at most $c_i$ items. This unit of flow should go towards *t* through one of the gadgets that are designed for agent *i* and each of the items. Furthermore, if *w* units of flow move through the gadget between $v_i$ and $v_a$, the min-cost solution for this flow is formed by using the edges $(v_{i, a, k}, v_a)$ for $k \in [w]$, and the cost of such a solution is equal to minus the utility that agent *i* gets from *w* copies of item *a*. This helps us find the optimal matching with the min-cost flow solution. The details of this reduction are presented in the rest of this section. Since the min-cost flow problem can be solved in polynomial time via linear programming (and also using various other algorithms [2]), we have the following property.

### Lemma 5.2

For capacity vector *C*, supply vector *M* and value matrix *V*, the min-cost flow instance defined in Definition 5.1 has an integral solution which we can find in polynomial time.

Our next lemma provides a connection between the solution of the min-cost flow instance of Definition 5.1 and the social welfare of the corresponding allocation for the instance of our problem.

### Lemma 5.3

The absolute value of the minimum-cost flow in *G*(*C*, *M*, *V*) is equal to the maximum social welfare of an allocation of items to agents with respect to values in *V*.

### Proof

Let $X^*$ be an allocation with the maximum social welfare w.r.t. *V*, and *F* be a min-cost flow in *G*(*C*, *M*, *V*). For $v, v' \in G$, let $F(v, v')$ be the flow from node *v* to node $v'$, and $\textsf{Cost}(F)$ the cost of *F*. We will show that $\textsf{sw}(X^*, V) = -\textsf{Cost}(F)$ by bounding $\textsf{Cost}(F)$ from above and below by $-\textsf{sw}(X^*,V)$.

To prove $\textsf{Cost}(F) \le -\textsf{sw}(X^*, V)$, we use $X^*$ to construct a flow $F^*$ as follows:

- $$F^*(v_i, v_{i, a, 1}) = x^*_i(a)$$ for $$i \in \mathcal {N}$$ and $$a \in \mathcal {M}$$;
- $$F^*(v_{i, a, j}, v_{i, a, j+1}) = x^*_i(a) - j$$ for $$j \in [x^*_i(a)-1]$$;
- $$F^*(v_{i, a, j}, v_a) = 1$$ for $$j \in [x^*_i(a)]$$;
- $$F^*(s, v_i) = \sum _{ a \in \mathcal {M}} x^*_i(a)$$ for $$i \in \mathcal {N}$$;
- $$F^*(v_a, t) = \sum _{ i \in \mathcal {N}} x^*_i(a)$$ for each $$a \in \mathcal {M}$$.

Observe that these flows satisfy the capacities of the edges, and thus $F^*$ is valid. Since *F* is the cost-minimizing flow, we have

$$\begin{aligned} \textsf{Cost}(F)&\le \textsf{Cost}(F^*) \\&=\sum _{i \in \mathcal {N}} \sum _{a \in \mathcal {M}} -V_{i, a, 1} \cdot F^*(v_i, v_{i, a, 1}) +\sum _{i \in \mathcal {N}} \sum _{a \in \mathcal {M}}\sum _{j = 1}^{x^*_i(a)-1} (V_{i, a, j}-V_{i, a, j+1}) \cdot F^*(v_i, v_{i, a, 1}) \\&=\sum _{i \in \mathcal {N}} \sum _{a \in \mathcal {M}} -V_{i, a, 1} \cdot x^*_i(a) + \sum _{i \in \mathcal {N}} \sum _{a \in \mathcal {M}}\sum _{j = 1}^{x^*_i(a)-1} (V_{i, a, j}-V_{i, a, j+1}) \cdot (x^*_i(a) - j) \\&=\sum _{i \in \mathcal {N}} \sum _{a \in \mathcal {M}} -V_{i, a, x^*_i(a)} + \sum _{i \in \mathcal {N}} \sum _{a \in \mathcal {M}}\sum _{j = 1}^{x^*_i(a)-1} V_{i, a, j} ( (x^*_i(a) - j) - (x^*_i(a) - (j-1)) ) \\&=\sum _{i \in \mathcal {N}} \sum _{a \in \mathcal {M}} -V_{i, a, x^*_i(a)} + \sum _{i \in \mathcal {N}} \sum _{a \in \mathcal {M}}\sum _{j = 1}^{x^*_i(a)-1} -V_{i, a, j} \\&=\sum _{i \in \mathcal {N}} \sum _{a \in \mathcal {M}}\sum _{j = 1}^{x^*_i(a)} -V_{i, a, j} = -\textsf{sw}(X^*, V). \end{aligned}$$

Next, to prove $\textsf{Cost}(F) \ge -\textsf{sw}(X^*, V)$, we use *F* to construct an allocation *X* with $x_i(a) = F(v_i, v_{i, a, 1})$. Doing similar calculations as above, and since $X^*$ is the welfare-maximizing allocation, we now have that

$$\begin{aligned} \textsf{Cost}(F)&= \sum _{i \in \mathcal {N}} \sum _{a \in \mathcal {M}}\sum _{j = 1}^{x_i(a)} -V_{i, a, j} = -\textsf{sw}(X, V) \ge -\textsf{sw}(X^*, V). \end{aligned}$$

This completes the proof. $\square$

Note that if we had complete access to the utilities (rather than access to threshold approvals), we could use the same methods to find the optimal solution. The size of the graph depends on sum of the capacities of the agents and number of items, which means with general capacities the problem is pseudo-polynomial. However, with constant capacities, the graph will have $O(n^2)$ nodes and the problem is solvable in polynomial time. We are now ready to present our deterministic mechanism $g_t$, which is a generalization of the deterministic mechanism $f_t$ that we used for the one-sided matching setting.

### Definition 5.4

For $\delta > 1$ and $t \in [n]$, we define the threshold vector $\vec {\tau }= (\delta ^{-1}, \delta ^{-2}, \ldots , \delta ^{-t})$. The *deterministic generalized matching mechanism* $g_t$ uses this threshold vector $\vec {\tau }$ and gets as input a profile $\textbf{S}$, constant agent capacities $\{c_1, \ldots , c_n\}$, and constant item supplies $\{m_1, \ldots , m_m\}$. The mechanism defines the vector $C = \langle c_1, \ldots , c_n\rangle$, the vector $M = \langle m_1, \ldots , m_m\rangle$, and the matrix *V* as follows: For every $i \in \mathcal {N}$, $a\in \mathcal {M}$ and $j \in [\min \{c_i, m_a\}]$, if $(a, j) \in S_{i,k}$ for some $k \in [t]$, then the mechanism defines $V_{i, a, j} = \tau _k$; otherwise, if $(a, j) \not \in \bigcup _{k=1}^t S_{i, k}$, then it defines $V_{i, a, j} = 0$. The mechanism computes the solution of the min-cost flow instance defined in Definition 5.1 with input *C*, *M* and *V*. For each agent $i \in \mathcal {N}$ and item $a \in \mathcal {M}$ the flow from $v_i$ to $v_{i,a,1}$ in the computed solution is the number of copies of item *a* that agent *i* receives.

Before we bound the distortion of the mechanism, we prove a technical lemma similar to Lemma 4.4 which provides us with a lower bound on the social welfare of the allocation computed by the mechanism in relation to the optimal social welfare.

### Lemma 5.5

If there is an allocation with social welfare $\mathsf {s^*}$, then $g_t$ outputs an allocation with social welfare at least $\delta ^{-1}(\mathsf {s^*}- T\cdot \tau _t)$.

### Proof

Let $X^*$ be an optimal allocation. We have

$$\begin{aligned} \textsf{sw}(X^*) \le \sum _{i \in \mathcal {N}} \sum _{k \in [t]} \sum _{(a, j) \in S_{i, k}} u_i(a, j) + T \cdot \tau _t. \end{aligned}$$

Recall that, if $(a,j) \in S_{i,k}$ for some *k*, then $u_i(a, j) \le \delta \cdot V_{i, a, j}$. This implies

$$\begin{aligned} \textsf{sw}(X^*) \le \delta \cdot \sum _{i \in \mathcal {N}} \sum _{k \in [t]} \sum _{(a, j) \in S_{i, k}} V_{i, a, j} + T \cdot \tau _t. \end{aligned}$$

Consequently, with respect to *V*, $X^*$ has a social welfare of at least $\delta ^{-1} (\textsf{sw}(X^*) - T \cdot \tau _t)$. This is a lower bound on the social welfare of the allocation computed by $g_t$, since, by Lemma 5.3, this is at least the social welfare of the allocation with maximum social welfare with respect to *V*. $\square$

We are now ready to show the upper bound on the distortion of $g_t$.

### Theorem 5.6

For $t \in [T]$ and $\delta = \root t \of {2T}$, the distortion of the deterministic generalized matching mechanism $g_t$ is $O(c\cdot \root t \of {T})$, where $c = \max _{i \in \mathcal {N}}\{c_i\}$.

### Proof

The structure of the proof is very similar to that of Theorem 4.5. Let $X = g_t(\textbf{S})$ be the allocation computed by $g_t$ when given as input an arbitrary input profile $\textbf{S}$ that is induced by some consistent utility profile $\vec {u}$. Clearly, for any $i \in \mathcal {N}$, $a\in \mathcal {M}$, and $j \in [\min \{c_i,m_a\}]$, $u_i(a,j) \ge V_{i, a, j}$ since $V_{i, a, j}$ is defined as a lower bound on this utility. By this and Lemma 5.3, if the graph *G*(*C*, *M*, *V*) admits a flow of cost *W*, then the social welfare of the corresponding allocation is at least |*W*|.

We now argue that the mechanism outputs an allocation with social welfare at least $\delta ^{-1}/(2c)$. To show this, consider any agent *i*. Since $\tau _t = \delta ^{-t} = 1/(2T)$, the sum of the utilities of agent *i* for the pairs (*a*, *j*) that he does not include in any approval set is at most $T\times 1/(2T) = 1/2$. Moreover, by the definition of $\vec {\tau }$, $u_i(a, j) \le \delta \cdot V_{i, a, j}$ for every $(a,j) \in \bigcup _{k =1}^t S_{i,k}$, and thus, for each agent *i*, we have

2 $$\begin{aligned} \sum _{k \in [t]} \sum _{(a, j) \in S_{i, k}} u_i(a, j) \ge 1/2 \implies \sum _{k \in [t]} \sum _{(a, j) \in S_{i, k}} V_{i,a,j} \ge \delta ^{-1}/2. \end{aligned}$$

Next, we find a partial allocation $B^*$ with social welfare at least $\delta ^{-1}/(2c)$. To do so, we bundle all copies of each item together and assign them all to a single agent (up to the capacity of the agent). In particular, construct a bipartite graph $G'$ with a node $v_i$ for each agent $i \in \mathcal {N}$ and a node $z_a$ for each item $a \in \mathcal {M}$. There is an edge of weight $\max _{j \in [\min (c_i, m_a)]} V_{i, a, j}$ between $v_i$ and $z_a$. Equation 2 implies that

$$\begin{aligned} \sum _{k \in [t]} \sum _{(a, j) \in S_{i, k}} w(v_i, z_a) \ge {\delta ^{-1}\over 2c}. \end{aligned}$$

Furthermore, for each edge $$(v_i, z_a) $$ in this bipartite graph, $$w(v_i, z_a) > \tau _t = 1/(2T)$$. Hence, by Lemma 4.3, $$G'$$ has a matching of weight $$\min \{\delta ^{-1}/(2c), n/(2T)\}$$. Since

$$\begin{aligned} \frac{n}{2T} = \frac{n}{2 \sum _{i \in \mathcal {N}}c_i} \ge \frac{n}{2n c} = \frac{1}{2c} \ge \frac{\delta ^{-1}}{2c}, \end{aligned}$$

we finally conclude that assigning all copies of an item to its matched agent in $G'$ gives us a social welfare of at least $\delta ^{-1}/(2c)$.

By Lemma 5.5, since $T\cdot \tau _t = 1/2$, we also have a lower bound of $\delta ^{-1}(\mathsf {s^*}- 1/2)$ on the social welfare of the allocation *X* computed by the mechanism, where $\mathsf {s^*}$ is the optimal social welfare. So, overall, we have established that

$$\begin{aligned} \textsf{sw}(X) \ge \max \left\{ \delta ^{-1}/(2c), \delta ^{-1}(s^* - 1/2) \right\} , \end{aligned}$$

and the distortion is at most

$$\begin{aligned} \frac{2\delta \cdot s^*}{\max \left\{ 1/c, 2s^* - 1 \right\} }. \end{aligned}$$

If $s^* \ge 1$, then $2s^*-1 \ge s^*$, and hence the distortion is at most $2\delta \in O(\root t \of {T})$. Otherwise, if $s^* < 1$, then the distortion is at most $2\delta c \in O(c\cdot \root t \of {T})$. $\square$

In many applications, the capacities and the supplies are constants. In this case, we have that $T = \Theta (n) = \Theta (m)$ and the following result, which is tight given the corresponding lower bound in Section 3.

### Corollary 5.7

When the capacities and supplies are constant, for any $t \in [T]$, there is a deterministic mechanism with distortion $O(\root t \of {n})$.

We also generalize our randomized mechanism to achieve a slightly better distortion bound.

### Definition 5.8

The * generalized randomized matching mechanism* $GR_t$ works as follows: With probability 1/2, it bundles the copies of each item together and selects a matching of the items to the agents uniformly at random; once the matching has been chosen, it assigns all copies of an item to its matched agent, subject to capacity and supply constraints. With the remaining 1/2 probability, it runs the deterministic mechanism $g_t$ with threshold vector $\vec {\tau }= (\delta ^{-1}, \delta ^{-2}, \ldots , \delta ^{-t})$ for $t \in [n]$ and some $\delta > 1$.

The proof of the next theorem follows along the lines of the proof of Theorem 4.8.

### Theorem 5.9

For $t \in [T]$ and $\delta = \root t+1 \of {2T}$, the distortion of the generalized randomized matching mechanism $GR_t$ is $O(c\cdot \root t+1 \of {T})$, where $c = \max \{n,m\}/n$.

### Proof

The structure of this proof is similar to that of Theorem 4.8. Let $X^*$ be an optimal allocation with social welfare $s^*$, and $X^2$ the allocation computed by the first part of the mechanism (the outcome of $g_t$).

In the first part of the mechanism (where a random matching between agents and items is chosen with probability 1/2), each agent is matched to each item with probability at least $1/\max \{n,m\}$. Indeed, fix an agent *i* and an item *a*. If $n\ge m$, there are ${n \atopwithdelims ()m} \cdot m!$ different matchings of items to agents, and *i* gets *a* in ${n-1 \atopwithdelims ()m-1}\cdot (m-1)!$ of them, leading to a probability of

$$\begin{aligned} \frac{{n-1 \atopwithdelims ()m-1}\cdot (m-1)!}{{n \atopwithdelims ()m} \cdot m!} = \frac{{n-1 \atopwithdelims ()m-1}}{\frac{n}{m}\cdot {n-1 \atopwithdelims ()m-1} \cdot m} = \frac{1}{n}. \end{aligned}$$

Otherwise, if $m > n$, there are ${m \atopwithdelims ()n} \cdot n!$ different matchings, and *i* gets *a* in ${m-1 \atopwithdelims ()n-1} \cdot (n-1)!$ of them, leading to a probability of

$$\begin{aligned} \frac{{m-1 \atopwithdelims ()n-1} \cdot (n-1)!}{{m \atopwithdelims ()n} \cdot n!} = \frac{{m-1 \atopwithdelims ()n-1}}{\frac{m}{n} \cdot {m-1 \atopwithdelims ()n-1} \cdot n} = \frac{1}{m}. \end{aligned}$$

Since the sum of the utilities of each agent when given all items up to capacity is 1, the expected social welfare from the first part is at least

$$\begin{aligned} \frac{1}{2} \sum _{i \in \mathcal {N}} \sum _{a \in \mathcal {M}} \frac{1}{\max \{n,m\}} \cdot u_i^+(a,\min \{c_i,m_a\}) = \frac{n}{2\cdot \max \{n,m\}}. \end{aligned}$$

For the second part (where the deterministic mechanism $g_t$ using $\vec {\tau }$ is employed), since $s:= T \cdot \tau _t = T \cdot \delta ^{-t} = \root t+1 \of {T}$, by Lemma 5.5, $g_t$ outputs an allocation with social welfare at least $\delta ^{-1}(\mathsf {s^*}- s)$, and thus the expected social welfare of the mechanism from the second part is at least $\frac{1}{2} \cdot \delta ^{-1}(\mathsf {s^*}- s)$.

Overall, we have established that

$$\begin{aligned} {{\,\mathrm{\mathbb {E}}\,}}_{X \sim GR_t(\textbf{S})}[\textsf{sw}(X)]&\ge \frac{n}{2\cdot \max \{n,m\}} + \frac{1}{2} \cdot \delta ^{-1}(\mathsf {s^*}- s) \\&\ge \frac{1}{2} \cdot \max \bigg \{ \frac{n}{\max \{n,m\}}, \delta ^{-1}(\mathsf {s^*}- s) \bigg \}, \end{aligned}$$

and thus the distortion is at most

$$\begin{aligned} \frac{2 \cdot \mathsf {s^*}}{\max \left\{ \frac{n}{\max \{n,m\}}, \delta ^{-1}(\mathsf {s^*}- s)\right\} }. \end{aligned}$$

If $\mathsf {s^*}\ge 2s$, then $\mathsf {s^*}- s \ge \mathsf {s^*}/2$ and the distortion is at most

$$\begin{aligned} \frac{2 \cdot \mathsf {s^*}}{\delta ^{-1}\mathsf {s^*}/2} = 4\delta = 4\root t+1 \of {T}. \end{aligned}$$

Otherwise, if $\mathsf {s^*}< 2s$, the distortion is at most

$$\begin{aligned} 2\cdot \frac{\max \{n,m\}}{n}\cdot \mathsf {s^*}\le \frac{4\max \{n,m\}}{n}\cdot \root t+1 \of {T}. \end{aligned}$$

In any case, the distortion is $O(c \cdot \root t+1 \of {T})$, where $c = \max \{n,m\}/n$. $\square$

Again, when the capacities and the supplies are constant, since the total capacity is of the same magnitude as the total supply, it follows that *n* and *m* are also of the same magnitude. Hence, the parameter $c = \max \{n,m\}/n$ is a constant and $T=\Theta (n)=\Theta (m)$, giving us the following result, which is again tight due to the corresponding lower bound from Section 3.

### Corollary 5.10

When the capacities and supplies are constant, for any $t \in [T]$, there is a randomized mechanism with distortion $O(\root t+1 \of {n})$.

### Remark 5.11

In the generalized setting that we considered in this section, the agents are allowed to receive potentially all available copies of the items, up to their capacity. However, in several applications, we might want to disallow this and set a limit $\ell _{i,a}$ on the number of copies of $a \in \mathcal {M}$ that agent $i \in \mathcal {N}$ can get. For example, in the paper assignment problem, each agent must be given at most one copy of each item since it does not make sense for someone to review a paper more than once. Such constraints can be handled in several ways. One of them is via the utility functions of the agents which, for scenarios like these, would simply assign a marginal value of 0 for any extra copy that exceeds the limit, that is, $u_i(a,j)=0$ for every $j > \ell _{i,a}$. If the utility function is not naturally defined this way, we can modify the min-cost flow instance by setting $V_{i, a, j} = -\infty$ for $j > \ell _{i, a}$, or by removing the corresponding edges in the graph.

## Conclusion and open problems

In this paper, we showed tight bounds on the best possible distortion of (both deterministic and randomized) mechanisms for matching settings (that capture important applications, including the one-sided matching problem and the paper assignment problem) when the given information about the preferences of the agents is of the form of threshold approvals.

Going forward, it would be interesting to explore whether improved tradeoffs can be achieved by using randomization not only during the decision phase of the mechanism but also for the definition of the threshold values, similarly to the works of [11] and [12]. Furthermore, one could explore other settings in which the same type of elicitation method can be applied, including various voting settings where the full potential of using multiple (as opposed to just one) threshold approvals has not been considered before, as well as other resource allocation settings, such as metric matching (which would correspond to the case where the items are chores rather than goods). These would also be interesting to study in combination with other constraints, such as truthfulness or fairness.

## Data Availability

No datasets were generated or analysed during the current study.

## Footnotes

1 We remark that without any normalization assumption, the distortion of deterministic mechanism can be shown to be unbounded in cases where we cannot distinguish between agents that have very large and very small utilities. In addition, normalization is necessary for the elication of threshold approval sets to be meaningful and provide useful information. See the paper of [10] for further motivation and justification of normalization assumptions.

2 Our results hold even when $\sum _{i \in \mathcal {N}} c_i = \Theta (\sum _{a \in \mathcal {M}} m_a)$, in which case we would need to define *T* as the maximum between these two quantities.
